# Prediction and causal inference of cardiovascular and cerebrovascular diseases based on lifestyle questionnaires

**DOI:** 10.1038/s41598-024-61047-w

**Published:** 2024-05-07

**Authors:** Riku Nambo, Shigehiro Karashima, Ren Mizoguchi, Seigo Konishi, Atsushi Hashimoto, Daisuke Aono, Mitsuhiro Kometani, Kenji Furukawa, Takashi Yoneda, Kousuke Imamura, Hidetaka Nambo

**Affiliations:** 1 School of Electrical Information Communication Engineering, College of Science and Engineering, Kanazawa University, Kanazawa, Japan; 2 Institute of Liberal Arts and Science, Kanazawa University, Kanazawa, Japan; 3 Department of Health Promotion and Medicine of the Future, Kanazawa University, Kanazawa, Japan; 4 Health Care Center, Japan Advanced Institute of Science and Technology, Nomi, Japan; 5 Faculty of Electrical, Information and Communication Engineering, Institute of Science and Engineering, Kanazawa University, Kanazawa, Japan; 6 Institute of Transdisciplinary Sciences, Kanazawa University, Kanazawa, Japan

**Keywords:** Cardiology, Health care, Medical research

## Abstract

Cardiovascular and cerebrovascular diseases (CCVD) are prominent mortality causes in Japan, necessitating effective preventative measures, early diagnosis, and treatment to mitigate their impact. A diagnostic model was developed to identify patients with ischemic heart disease (IHD), stroke, or both, using specific health examination data. Lifestyle habits affecting CCVD development were analyzed using five causal inference methods. This study included 473,734 patients aged ≥ 40 years who underwent specific health examinations in Kanazawa, Japan between 2009 and 2018 to collect data on basic physical information, lifestyle habits, and laboratory parameters such as diabetes, lipid metabolism, renal function, and liver function. Four machine learning algorithms were used: Random Forest, Logistic regression, Light Gradient Boosting Machine, and eXtreme-Gradient-Boosting (XGBoost). The XGBoost model exhibited superior area under the curve (AUC), with mean values of 0.770 (± 0.003), 0.758 (**± **0.003), and 0.845 (**± **0.005) for stroke, IHD, and CCVD, respectively. The results of the five causal inference analyses were summarized, and lifestyle behavior changes were observed after the onset of CCVD. A causal relationship from ‘reduced mastication’ to ‘weight gain’ was found for all causal species theory methods. This prediction algorithm can screen for asymptomatic myocardial ischemia and stroke. By selecting high-risk patients suspected of having CCVD, resources can be used more efficiently for secondary testing.

## Introduction

Cardiovascular and cerebrovascular diseases (CCVD) are major causes of death in the Japanese population^1,2^. These diseases are classified as ‘symptomatic’ or ‘asymptomatic,’ according to the presence or absence of symptoms. Prevention, early diagnosis, and treatment are important to prevent poor quality of life (QoL) and death^2–4^. In some cases, such as stroke, early treatment may lead to the recovery of neurological functions. Lifestyle modification is an important component of such prevention. Appropriate diet, moderate exercise, and good sleep management can reduce the risk of CCVD. Health check-ups are also crucial. In Japan, specific health checkups focusing on metabolic syndrome have been conducted since 2008 to prevent lifestyle-related diseases^5^. Based on the results of these checkups, specific health guidelines are provided to review lifestyle habits pertaining to exercise, diet, and smoking.

In recent years, artificial intelligence (AI) has attracted significant attention because of its use in preventive medicine. AI methods can automatically identify important patterns in an individual’s clinical data and predict disease onset and prognosis^6,7^. Several studies have demonstrated the high accuracy of AI models for stroke and coronary artery disease^8,9^. Investigating causal inference in observational studies is also an effective method, as it captures real-world events and behaviors and may provide results that resemble real-world situations compared to experimental studies^10,11^.

Several predictive models for CCVD have been proposed, but reports are limited. In addition, there are no reports examining causal relationships among lifestyle factors used for prediction. Therefore, this study aimed to examine how specific lifestyle habits affect the CCVD risk by testing multiple causal relationships. In this study, we developed a predictive model for CCVD using data obtained from metabolic syndrome screening in the general population.

## Results

### Participant characteristics

A total of 473,734 participants who underwent medical examinations were included in the KMA database. 261,645 individuals were excluded from the dataset because they had at least one missing value. The exclusion criteria were applied, and the remaining patients were classified into stroke (n = 10,713), IHD (n = 20,922), CCVD (n = 3868), and normal (n = 176,586) groups. Table 1 lists the baseline characteristics of each disease and normal group. The IHD, stroke, and CCVD groups showed significant differences in age, female sex ratio, BMI, prevalence of HT, prevalence of DM, prevalence of DL, SBP, DBP, HbA1c, TG, HDL-C, and T-Cho compared to the normal group. Table 2 lists the lifestyle behaviors of the participants based on the questionnaire. There were significant differences in all lifestyle behaviors in the IHD, stroke, and CCVD groups compared with those in the normal group (*P* < 0.05, vs. normal group).

**Table 1:** Clinical characteristics of participants who underwent community screening in Kanazawa city.

	Normal	IHD	Stroke	CCVD
N	176,586	20,922	10,713	3868
Age (years)	69 ± 11	76 ± 9	75 ± 9	77 ± 8
Female (%)	101,765 (57.6)	9159 (43.8)	4086 (38.1)	1320 (34.1)
BMI (kg/m^2^)	22.7 ± 3.2	23.2 ± 3.3	23.3 ± 3.3	23.5 ± 3.4
Hypertension (%)	66,386 (37.6)	12,240 (58.5)	6816 (63.6)	2647 (68.4)
Diabetes (%)	17,289 (9.8)	3299 (15.8)	1795 (16.8)	882 (22.8)
Dyslipidemia (%)	39,433 (22.3)	6319 (30.2)	2763 (25.8)	1099 (28.4)
SBP (mmHg)	128 ± 17	128 ± 16	130 ± 15	129 ± 16
DBP (mmHg)	74 ± 10	72 ± 1	74 ± 11	71 ± 11
HbA1c (%)	5.6 ± 0.6	5.7 ± 0.7	5.7 ± 0.7	5.8 ± 0.8
TG (mg/dL)	120 ± 80	123 ± 77	125 ± 71	124 ± 73
HDL-Cho (mg/dL)	61 ± 16	57 ± 15	56 ± 15	53 ± 15
T-Cho (mg/dL)	201 ± 34	188 ± 34	190 ± 32	181 ± 33

Data are presented as the mean (SD) or number of participants (%).

*BMI* body mass index, *CCVD* cardiovascular and cerebrovascular diseases, *DBP* diastolic blood pressure, *HbA1c* hemoglobin A1c, *HDL-C* high-density lipoprotein cholesterol, *IHD* ischemic heart disease, *SBP* systolic blood pressure, *T-Cho* total cholesterol, *TG* triglycerides.

*P < 0.05, vs normal group.

**Table 2:** Lifestyle behaviors of participants.

	Normal	IHD	Stroke	CCVD
N	176,586	20,922	10,713	3868
Current smoker	22,288 (12.6)	2068 (9.9)	1215 (11.3)	325 (8.4)
Weight gain	48,947 (27.7)	6713 (32.1)	3535 (33.0)	1434 (37.1)
Regular exercise	77,125 (43.7)	9142 (43.7)	4378 (40.9)	1431 (37.0)
Daily activity	98,332 (55.7)	11,712 (56.0)	5378 (50.2)	1782 (46.1)
Walking speed	83,433 (47.2)	8200 (39.2)	3399 (31.7)	1125 (29.1)
Chewing				
Chew everything	49,497 (28.0)	6196 (29.6)	3185 (29.7)	1215 (31.4)
Sometimes difficult to chew	126,898 (71.9)	14,676 (70.1)	7501 (70.0)	2632 (68.0)
Hardly chew anything	191 (0.1)	50 (0.2)	27 (0.3)	21 (0.5)
Diet habits				
Eating speed				
Fast	48,634 (27.5)	5516 (26.4)	2537 (23.7)	917 (23.7)
Normal	105,645 (59.8)	11,674 (55.8)	5854 (54.6)	2087 (54.0)
Slow	22,307 (12.6)	3732 (17.8)	2322 (21.7)	864 (22.3)
Frequent skipping of breakfast	13,899 (7.9)	1468 (7.0)	784 (7.3)	313 (8.1)
Eating dinner late	32,010 (18.1)	3826 (18.3)	2286 (21.3)	894 (23.1)
Frequent snacking				
Everyday	27,080 (15.3)	2981 (14.2)	1503 (14.0)	553 (14.3)
Occasional	142,261 (80.6)	17,024 (81.4)	8732 (81.5)	3160 (81.7)
Rare	7245 (4.1)	917 (4.4)	478 (4.5)	155 (4.0)
Drinking habits				
Frequency of alcohol drinking				
Everyday	63,452 (35.9)	7839 (37.5)	3948 (36.9)	1330 (34.4)
Occasional	49,090 (27.8)	5101 (24.4)	2572 (24.0)	866 (22.4)
Rare	64,044 (36.3)	7982 (38.2)	4193 (39.1)	1672 (43.2)
Alcohol consumption				
< 1 Glass of sake	127,592 (72.3)	15,259 (72.9)	7851 (73.3)	2870 (74.2)
1–2 Glass of sake	33,738 (19.1)	4046 (19.3)	2041 (19.1)	721 (18.6)
2–3 Glass of sake	11,819 (6.7)	1268 (6.1)	629 (5.9)	207 (5.4)
≥ 3 Glass of sake	3437 (19.5)	349 (1.7)	192 (1.8)	70 (1.8)
Sleep habits	139,365 (78.9)	16,354 (78.2)	8639 (80.60	3087 (79.8)

Data are presented as number of participants (%).

*CCVD* cardiovascular and cerebrovascular diseases, *IHD* ischemic heart disease.

*P < 0.05, vs normal group.

### Feature importance ranking

Supplementary Figure 1 shows the feature importance ranking for each prediction model using Dataset 2. eGFR, PG, and TG were consistently among the top 10 important features in all four IHD prediction models. MCV and TG were consistently among the top 10 important features in all four stroke prediction models. ALT, Hb, and TG were consistently among the top 10 important features in all four CCVD prediction models. TG consistently ranked in the top 10 important features in all 12 models.

Supplementary Figure 2 depicts the variation in the AUC as features were incrementally added to each prediction model, starting with the highest-ranked feature. For the LGBM, 19 features were chosen for stroke, 17 for IHD, and 20 for CCVD. For the RF model, 19, 15, and 20 features were selected for the stroke, IHD, and CCVD, respectively. In the XGBoost model, 19, 16, and 19 features were selected for the stroke, IHD, and CCVD, respectively. Finally, in the LR model, stroke incorporated four features, IHD incorporated 18 features, and CCVD incorporated two.

### Predictive performance

**Figure 1:**
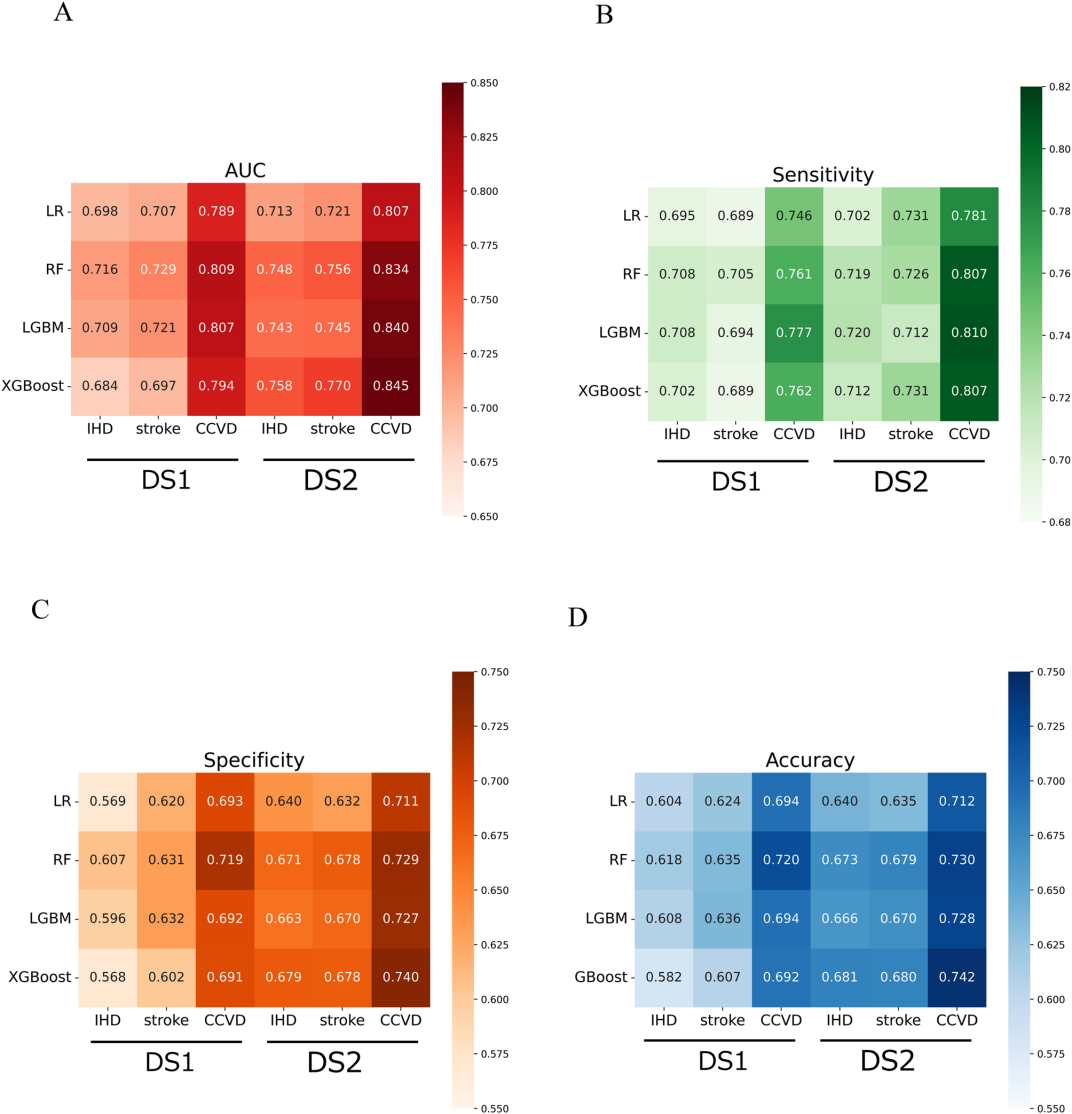
Performance of disease prediction algorithms. Heatmap comparing the area under the receiver operating characteristic curve (**A**), sensitivity (**B**), specificity (**C**), and accuracy (**D**). Counts in each box represent an average of 20 counts. The data set and model combination with the highest AUC and Accuracy was XGBoost in data set 2 for IHD, stroke, and CCVD. The highest sensitivity was for LGBM in Data Set 2 for IHD, for LR and XGBoost in Data Set 2 for stroke, and for LGBM in Data Set 2 for CCVD. The data set and model combination with the highest specificity was XGBoost from Data Set 2 for IHD and CCVD, and RF or XGBoost from Data Set 2 for stroke. *LR* logistic regression, *RF* random forest, *LGBM* light gradient boosting machine, *XGBoost* eXtreme gradient boosting, *IHD* ischemic heart disease, *CCVD* cardiovascular and cerebrovascular diseases, *DS1* dataset 1, *DS2* dataset 2.

Figure 1 shows the performance metrics of the CCVD predictive models. For Dataset 1, the RF models consistently achieved the highest AUCs, with mean values of 0.729 (SD 0.003), 0.716 (SD 0.003), and 0.809 (SD 0.005) for stroke, IHD, and CCVD, respectively.

In comparison, when Dataset 2 was analyzed, the XGBoost model exhibited the best AUC. The mean AUCs were 0.770 (SD = 0.003), 0.758 (SD = 0.003), and 0.845 (SD = 0.005) for stroke, IHD, and CCVD, respectively. The prediction model employing Dataset 2 surpassed that employing Dataset 1 for all the accuracy metrics.

### Causal inference

Supplementary Figure 3 illustrates the causal network and inferred directions of causality determined using the five causal search methodologies. Supplementary Table 1 shows the score rankings for the lifestyle-related features. First place was “walking speed” with a score of 142. Second place went to “chewing” with a score of 100, and third was “weight gain” with a score of 85. In fourth place were “sleep habits” and “regular exercise” with the same score of 84. The top five rankings were selected as the features for causal inference. The Direct LiNGAM technique visually represents the magnitude of influence of one variable on another using a partial regression coefficient indicated by a connecting arrow. For the three models utilizing NOTEARS, causal arrow strength is denoted as ‘Edge Weight’. However, Bayesian networks do not quantitatively illustrate causality through metrics, such as partial regression coefficients or edge weights.

**Figure 2:**
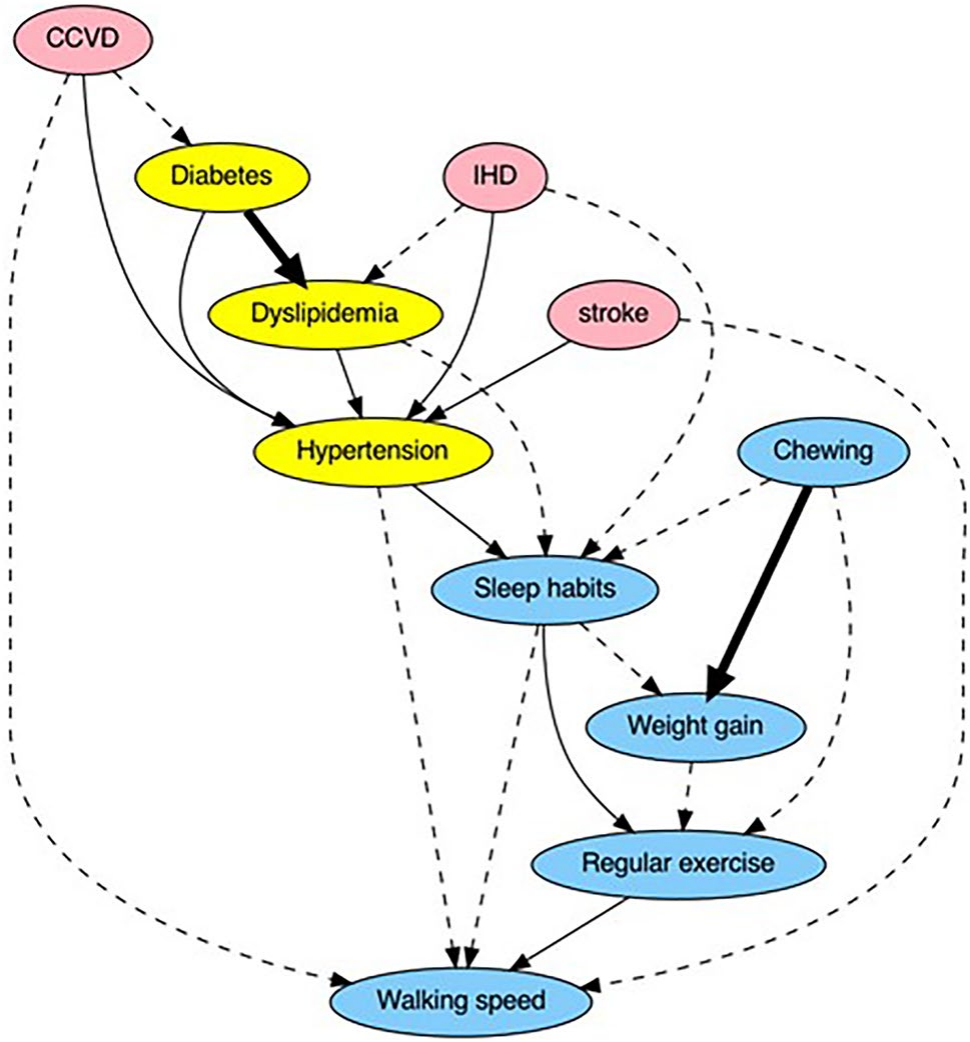
Ensemble causal inference. Analyses were performed for each of the five causal inference models—Direct LiNGAM, NOTESRS, NOTESRS with Lasso, NOTESRS with PyTorch, and Bayesian networks—and the results were integrated. Three of the five models that matched the causal directions are shown as dotted lines, four as solid lines, and five as bold lines. Causal direction indicated by all five causal inference models (bold arrows): the onset of diabetes is causally related to the incidence of dyslipidemia. Decreased chewing ability is associated with increased weight compared to weight around age 20. Causal direction indicated by four of the five causal inference models (solid arrows): Hypertension is causally related to the development of CCVD, IHD, Stroke, diabetes, and dyslipidemia. The development of hypertension is causally related to not getting enough rest with sleep. When people do not get enough sleep, they do not implement a regular exercise routine of at least 30 min twice a week, resulting in a lower walking speed than the average for the same age group of the same sex. *IHD* ischemic heart disease, *CCVD* cardiovascular and cerebrovascular disease. Weight gain: Have you gained 10 kg or more since turning 20 kg? Regular exercise: Do you engage in at least 30 min of exercise twice a week for a minimum of 1 year? Walking speed: Do you walk faster than your peers? Chewing: How thoroughly do you chew food during meals? Sleep habits: Do you feel that you get sufficient sleep?

Figure 2 presents the ensemble analysis results and summarizes the findings of the five causal inference methods. Relationships identified as causal by three or more methods are indicated by arrows and are differentiated using dashed, solid, or bold lines. Importantly, the causal link from DM to DL and the pathway from ‘chewing’ to ‘weight gain’ were consistently supported across all five techniques.

## Discussion

A predictive model including lifestyle questionnaires was developed to diagnose CCVD. There are several reports on cardiovascular disease and stroke prediction models that use lifestyle information^12–14^. Li et al. developed several models to predict cardiovascular disease using a dataset including lifestyle-related items such as smoking, alcohol consumption, dietary patterns, and physical activity for 1,887,710 adults in northwest China and found that the RF prediction model had the best prediction accuracy with an AUC of 0.723^12^. Zhu et al. found the best prediction accuracy for stroke, with an AUC of 0.686, in a dataset of 2147 members of the general population using a model that included sex, age, lifestyle habits, genetic factors, medical history, nasal examination results, and blood sampling results^14^. However, these studies did not examine causality between the features used for prediction.

The precise predictive results of the algorithm are expected to alert individuals and trigger behavioral changes that lead to further guidance and closer examination. Conversely, the algorithm can reduce the financial burden and time wasted by patients with a low predictive risk who would otherwise undergo secondary testing. However, caution should be exercised when implementing this algorithm, as the model does not predict the future development of the disease, but rather indicates the possibility of prior myocardial ischemia or stroke occurrence in health-screening participants who are unaware of their symptoms. Asymptomatic stroke does not present with symptoms such as paralysis or sensory disturbance^15^, whereas asymptomatic myocardial ischemia is characterized by few or no typical symptoms of IHD, such as chest pain, pressure, nausea, and shortness of breath^16^. In practice, these asymptomatic conditions are detected incidentally using electrocardiography, computed tomography, and magnetic resonance imaging (MRI). Moreover, asymptomatic stroke increases the risk of symptomatic stroke and vascular cognitive impairment^17–19^. Asymptomatic myocardial ischemia is more common in the elderly^20,21^ and patients with DM^22–25^, and is associated with increased cardiac and all-cause mortality, which is particularly important in the presence of other coronary disease risk factors^26–29^. A diagnosis of asymptomatic stroke or myocardial ischemia does not imply the need for aggressive invasive treatment. However, intensive treatment of lifestyle-related diseases should be considered^2,3^. Choosing patients with a high likelihood of asymptomatic disease for treatment is reasonable to prevent the future development of symptomatic CCVD and may be an economically advantageous approach owing to optimized target selection and prevention of unnecessary treatment costs. Further research on the practical efficiency and health economics of this algorithm is required.

The results of the causal inference, a novel element of this study, are surprising. We expected a causal relationship between worsening lifestyle habits and cerebrovascular diseases via lifestyle-related diseases, similar to the “metabolic domino” proposed by Ito et al.^30^. However, in the present study, behavior was observed to change after the onset of CVD. This result may be attributed to the change in participants’ health awareness after the onset of CCVD and the effectiveness of the National Health Guidance and Health Guidance System. When participants develop CCVD and become aware of its symptoms for the first time, they may become more conscious of the changes they need to make to prevent further exacerbation of the disease and try to improve their lifestyle. It is also not yet clear whether this system is an effective mechanism for encouraging participants to change their behavior for the primary prevention of CCVD.

Fukuma et al. reported no evidence that Japanese government-led national health and health guidance interventions were associated with improvements in cardiovascular risk factors among Japanese working-age men^31^. They evaluated changes in obesity status and cardiovascular risk factors (blood pressure, hemoglobin A1c levels, HDL cholesterol levels) 1 to 4 years after screening, but not stroke, IHD, or CCVD. However, if interventions do not improve cardiovascular risk factors, they will not prevent the development of CCVD either. General health screening programs in other countries have also been reported to be ineffective in reducing mortality from cardiovascular disease^32,33^. Inter9914 in Denmark reported that the incidence of IHD, stroke, and total mortality after 10 years was not significantly different between the control groups, even with interventions such as health checkups, lifestyle guidance for five years, and, if necessary, referral to a medical institution^33^. Ensemble causal analysis suggests that the primary prevention system for metabolic syndrome screening and specific health guidance in Japan has not fully achieved its objectives, such as preventing CCVD.

The ensemble causal network revealed significant relationships such as: (i) The causal relationship “the lower the chewing ability, the more weight gain” was found in all five causal inference models. Several studies have suggested that chewing slowly and often during meals is associated with a lower BMI^34–36^. Chewing well is an effective way to reduce the rate of eating and may contribute to a lower risk of obesity^37,38^. (ii) Sleep habits are frequently associated with diseases and other lifestyle habits in a causal manner. Short sleep duration and sleep disturbances are associated with adverse cardiometabolic risks such as obesity, HT, type 2 DM, and cardiovascular disease^39,40^. IHD is complicated by heart failure (HF). Approximately 75% of patients with heart failure experience sleep disturbance^41^. Obstructive sleep apnea, an obvious risk factor for heart disease, is associated with poor sleep quality, HT, and DL^42^. Thus, sleep may have multifaceted effects on the development of CCVD.

Finally, the observed variability in the causal inference results can be attributed to the occurrence of systematic biases due to confounding factors, such as selection and measurement^43^. Causal inference may be more susceptible to bias due to potential confounding factors when the sample size is small, resulting in variable causal relationships. Shimizu et al. reported a false causal association when analyzing a 1300-sample, six-feature dataset with potential confounding factors using Direct LiNGAM^44^. However, when the sample size of the dataset is large, the causal direction converges to the right in Direct LiNGAM^44,45^. There were 200,000 cases in this dataset, which may not have had a significant effect on causal direction. Additionally, a selection bias may have occurred in the population. Nakao et al. reported that those who participated in the health guidance intervention improved more dramatically in both weight and cardiovascular risk factors than those who did not participate^46^. Predictive models and causal relationships may differ significantly depending on the participation rate of health guidance interventions. The rate of specific health guidance provided in Kanazawa City ranged from 21.9 to 35.8%, while the national average implementation rate of specific health guidance was low, ranging from 7.74 to 23.2%^47^. These regional differences may affect the predictive accuracy and causality of the developed algorithms. Finally, lifestyle and medical history information relied on the participant’s responses to the questionnaire. Measurement bias may have existed if the participants misidentified and responded to their health information. Therefore, it is impossible to eliminate all biases when using real-world data. Therefore, we analyzed and integrated the results using five different methods to ensure their robustness in the presence of these variations. None of the models showed any variations in the results, indicating that CCVD affected lifestyle-related diseases or specific lifestyle habits. The ensemble causal network method was more reliable than the single-model causal inference method.

In conclusion, we established a predictive model for CCVD using data from lifestyle questionnaires, physical observations, medical histories, and general laboratory results obtained during health examinations. Using real-world data, we used an ensemble causal network to represent the causal relationships among lifestyle, lifestyle-related diseases, and CCVD. The algorithm can predict whether health checkup participants experience asymptomatic myocardial ischemia and stroke, which may lead to savings in healthcare costs through the efficient use of healthcare resources for secondary health checkups. However, the results of this causal inference should be interpreted with caution. The results should be analyzed in other populations, and the reproducibility of the causal relationships should be confirmed. Further research is required to determine the usefulness of this algorithm in the Japanese health checkup system.

## Materials and methods

### Study participants

This study design is a secondary data analysis using community health screening. The study included 473,734 participants aged 40 years or older who underwent community health screening in Kanazawa City between 2009 and 2018. Medical institutions in charge of health checkups were sent identical manuals in accordance with the guidelines of the respective associations and checkups were conducted accordingly. During the checkups, clinicians performed a standard consultation and recorded data on height, weight, waist circumference, blood pressure, biochemical test results, urinalysis, and lifestyle questionnaires^6^. The study was approved by the Ethics Committee of the Kanazawa Medical Association (KMA) (No. 16000003) and the Ethics Committee of Kanazawa University (No. 2019-080) and was conducted in accordance with the Declaration of Helsinki and ethical guidelines for human medical research. All data were anonymized. The Ethics Committee waived the need for informed consent, as this was secondary data use. An opt-out notification form regarding the study was provided on the KMA website (http://www.kma.jp/kenkyu/kenkyu_index.html).

### Features

The KMA database contains information on various clinical parameters as previously reported^6^. Six variables were collected from the dataset: age, sex, body mass index (BMI), waist circumference, systolic blood pressure (SBP), and diastolic blood pressure (DBP).

Laboratory blood parameters were measured within 24 h of collection using an automated clinical chemistry analyzer. These parameters included plasma glucose (PG), hemoglobin A1c (HbA1c), total cholesterol (T-Cho), triglycerides (TG), low-density lipoprotein cholesterol, high-density lipoprotein cholesterol (HDL-C), serum creatinine, estimated glomerular filtration rate (eGFR), serum uric acid, aspartate aminotransferase, alanine aminotransferase, gamma-glutamyl transpeptidase, white blood cell count, red blood cell count, hemoglobin, hematocrit, mean corpuscular volume (MCV), mean corpuscular hemoglobin, mean corpuscular hemoglobin concentration, and platelet count. The test procedures followed the specimen testing methods recommended by the Japanese Society for Clinical Chemistry.

Standardized questionnaire items for specific health checkups were developed by experts under the initiative of the Japanese Ministry of Health, Labor and Welfare. The questionnaire covered medical history including treatment for hypertension (HT), diabetes (DM), and dyslipidemia (DL). We also inquired whether the patient had previously experienced stroke, ischemic heart disease (IHD), chronic kidney disease (CKD), or anemia. The questionnaire comprised 13 lifestyle-related questions. The details of the questionnaire are presented in Supplementary Table 2.

### Dataset construction

Two datasets were assembled to develop a predictive model based on questionnaire responses. Dataset 1 comprised data from 13 lifestyle-related questionnaire items in addition to age, sex, BMI, waist circumference, SBP, and DBP. Dataset 2 consisted of blood and urine test results from Dataset 1. The definitions of predictive brain disease, IHD, and complications associated with both brain diseases and IHD were derived from a questionnaire.

### Statistical analysis for clinical background

Data are expressed as mean (SD) or percentage. The clinical background of each disease group was compared with that of the normal group. Normality was assessed using the Shapiro–Wilk test. Normally distributed data with equal variances were compared using the Student’s t-test, whereas data with unequal variances were compared using Welch’s t-test. *P* < 0.05 was considered significant. Statistical analyses were performed using Python 3.8.3 programming language (Python Software Foundation, Wilmington, DE, USA), and SciPy 1.5.2.

### General process of prediction model construction

**Figure 3:**
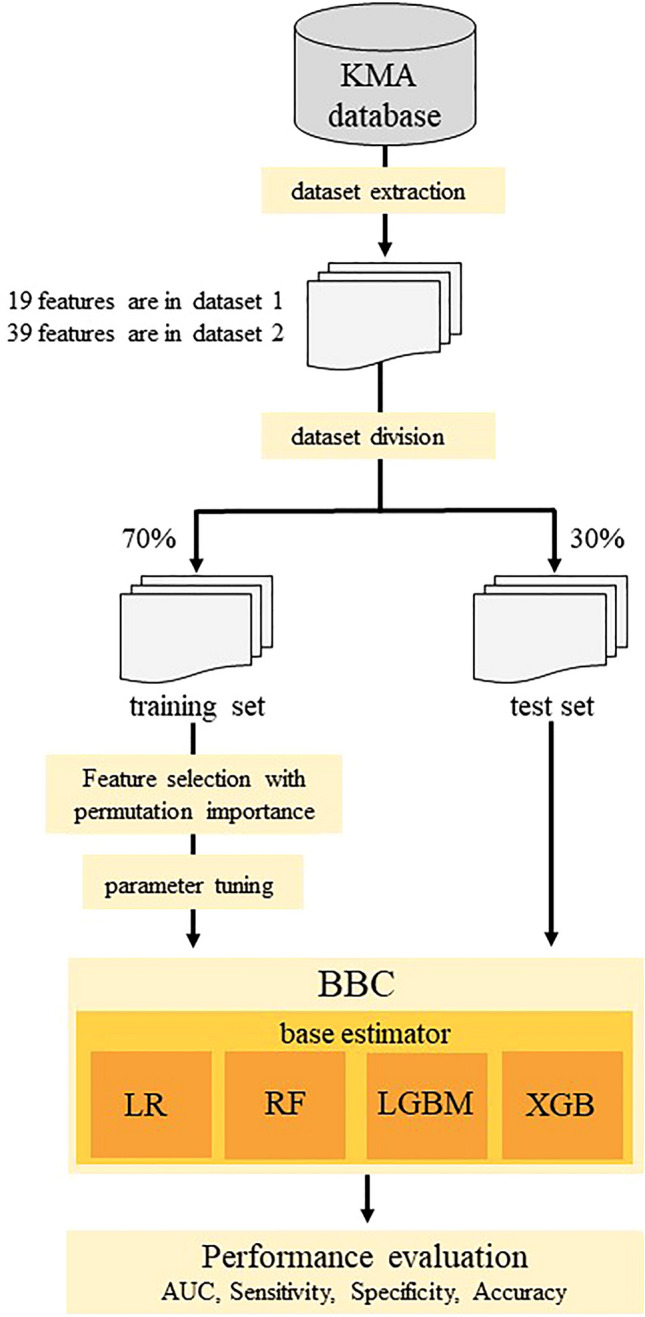
Procedure for developing a diagnostic prediction algorithm. Of the participants enrolled in the KMA database, 261,645 participants with missing data were excluded. Participants were then classified into stroke group (n = 10,713), IHD group (n = 20,922), CCVD group (n = 3868), and normal group (n = 176,586). Dataset 1 consisted of 19 features including age, gender, BMI, waist circumference, SBP, DBP, and 13 lifestyle-related questions. Dataset 2 consisted of 19 variables from dataset 1 and 20 variables including blood tests. The cases were split into training and test data in a 7:3 ratio. The machine learning algorithms (MLAs) used as base estimators were Logistic regression (LR), Random Forest (RF), Light Gradient Boosting Machine (LGBM), and eXtreme-Gradient-Hyperparameter tuning and feature selection by permutation importance were used for each model to optimize MLA performance. A Balanced Bagging Classifier was used to address the imbalance in the number of cases in the CCVD and healthy groups. The prediction performance was evaluated using the area under the receiver operating characteristic curve (AUC), sensitivity, specificity, and accuracy.

The procedure for building and predicting the CCVD model is illustrated in Fig. 3. Patients with missing data were excluded. The data set was assembled using a stratified extraction method to split the data in a 7:3 ratio for training and testing while maintaining the distribution of it.

The machine learning algorithms used were Light Gradient Boosting Machine (LGBM)^48^, Random Forest (RF)^49^, Logistic regression (LR)^50^, and eXtreme-Gradient-Boosting (XGBoost)^51^. Hyperparameter tuning and feature selection by permutation importance were used for each model to optimize the performance of the machine learning models. Parameter tuning is the process of adjusting external configuration values that control the model’s learning process to maximize the model’s predictive performance. Permutation importance is a method to evaluate the importance of a feature. We randomly swapped the values of certain features, evaluated how they affected the performance of the model, and ranked the features in order of importance. Based on the ranked list of features, the minimum set of features needed to maintain or improve the model’s performance is selected. To address the imbalance in the number of cases in the CCVD and healthy groups, a Balanced Bagging Classifier was used. The Balanced Bagging Classifier is a method that combines bagging and under sampling, and used LGBM, RF, LR, or XGBoost as the base estimator. Youden Index was used to determine the cutoff value for the receiver operating characteristic (ROC) curve. The model-construction process was repeated 20 times. Predictive metrics, such as accuracy, area under the ROC curve (AUC), sensitivity, and specificity, were computed and averaged. For models utilizing Dataset 2, we gauged the significance of the clinical tests via permutation importance^52^ and selected the features exhibiting the highest AUC^53^.

### Construction and integration of causal inference

Causal inferences were drawn using the Direct Linear Non-Gaussian Acyclic Model (LiNGAM)^44^, Non-combinatorial Optimization via Trace Exponential and Augmented Lagrangian for structure learning (NOTEARS)^54^, NOTEARS with the least absolute shrinkage and selection operator^54^, NOTEARS with PyTorch^54^, and a Bayesian network^55,56^. For causal inference, the variables incorporated were CCVD, stroke, IHD, DM, HT, DL, and lifestyle factors. Features were selected based on their importance, and the top five features were identified using permutation importance. The 13 lifestyle items were selected as follows:

1. Determining feature importance using permutation importance.
2. The questionnaire items were ranked from 1st to 13th position and scored in descending order: 13 points for 1st place, 12 points for 2nd place, 2 points for 12th place, and 1 point for 13th place.
3. Scoring was repeated 12 times across the four prediction models and three diseases to achieve cumulative scores. Cumulative scores determined the top five lifestyle items incorporated into the causal inference.

All features used for causal inference were standardized and used in the analysis.

### Supplementary Information

Supplementary Figure S1.

Supplementary Figure S2.

Supplementary Figure S3.

Supplementary Information.

Supplementary Tables.

## Data Availability

De-identified participant data were shared. Please contact Shigehiro Karashima at skarashima@staff.kanazawa-u.ac.jp. The data will be restricted by the Kanazawa University IRB depending on the intended use. The data were shared in an Excel file after approval by the Kanazawa University Institutional Review Board.

## Code availability

The code used for the analysis was shared at the following link. https://zenodo.org/records/10703345.
