# Supplementary figures and images for: Prediction and causal inference of cardiovascular and cerebrovascular diseases based on lifestyle questionnaires
Source: Sci Rep. 2024 May 7;14:10492. doi: 10.1038/s41598-024-61047-w (PMC11076536; doi:10.1038/s41598-024-61047-w)

Supplementary Figure 1

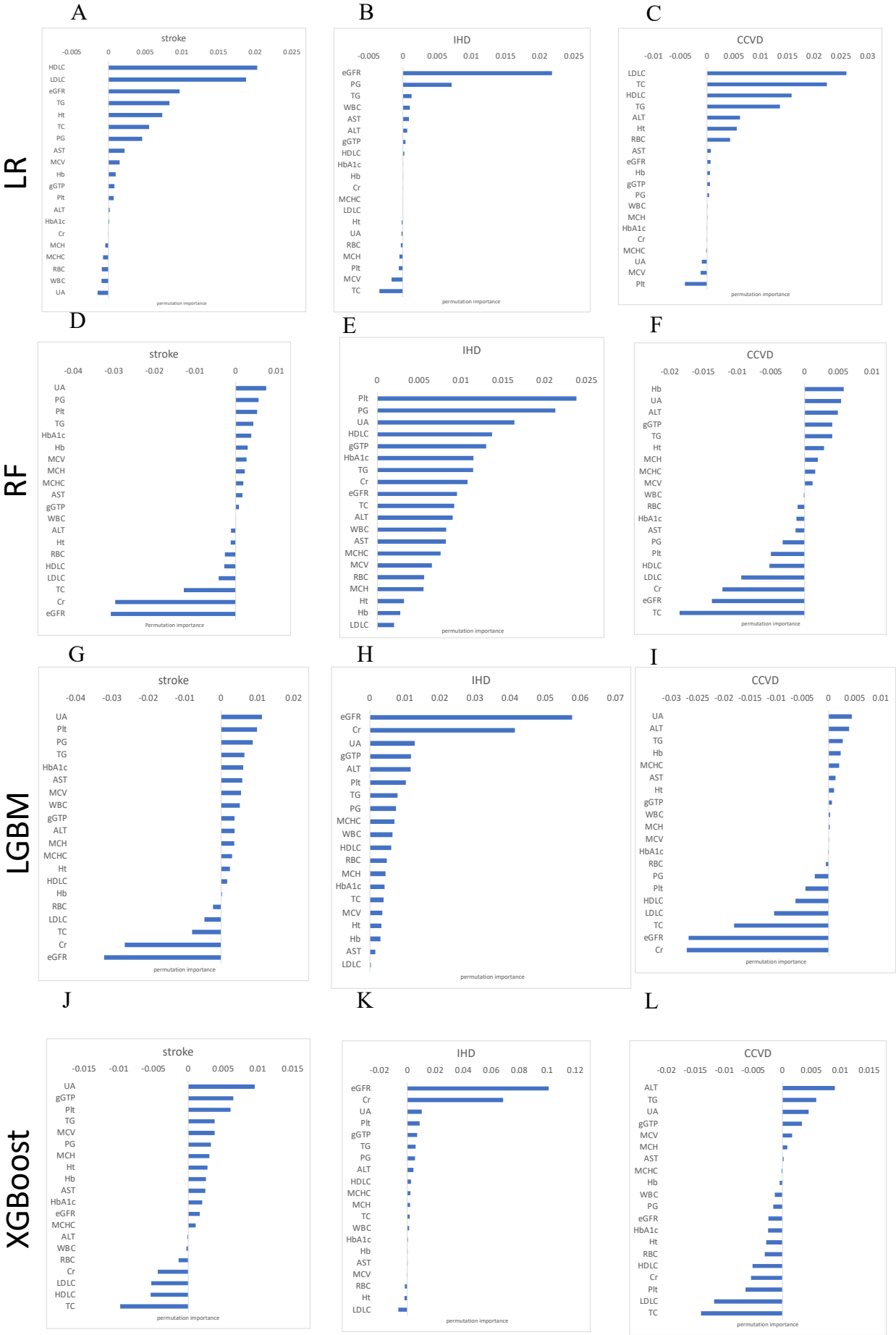

Supplement: Supplementary file 1 — Supplementary Figure S1. [file 41598_2024_61047_MOESM1_ESM.pdf]

Supplementary Figure 2

A

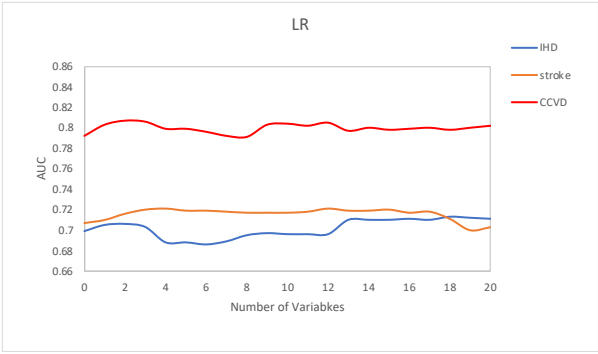

B

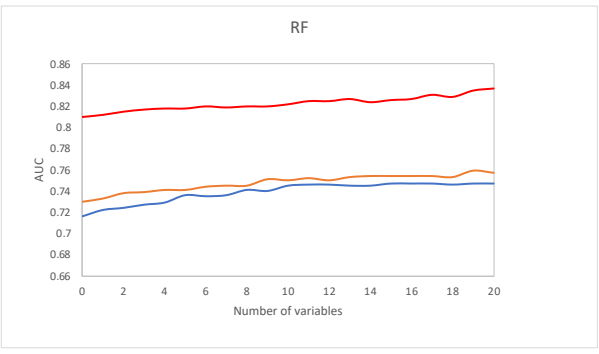

C

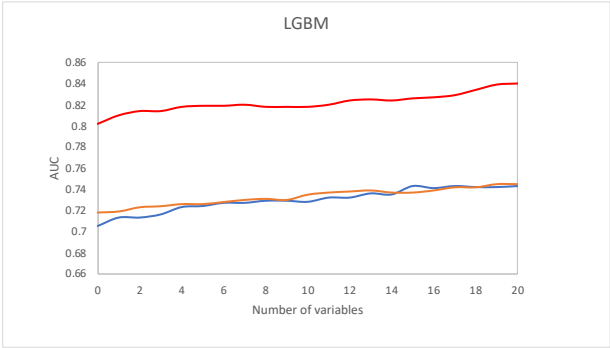

D

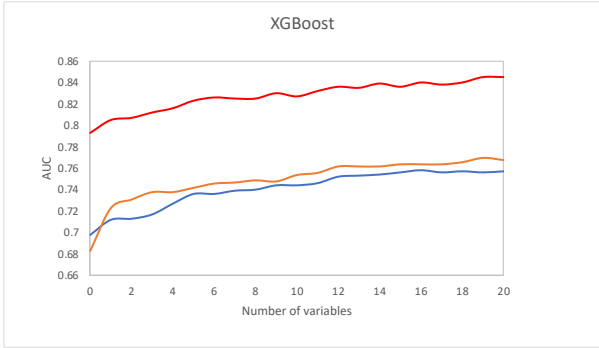

Supplement: Supplementary file 2 — Supplementary Figure S2. [file 41598_2024_61047_MOESM2_ESM.pdf]

Supplementary Figure 3

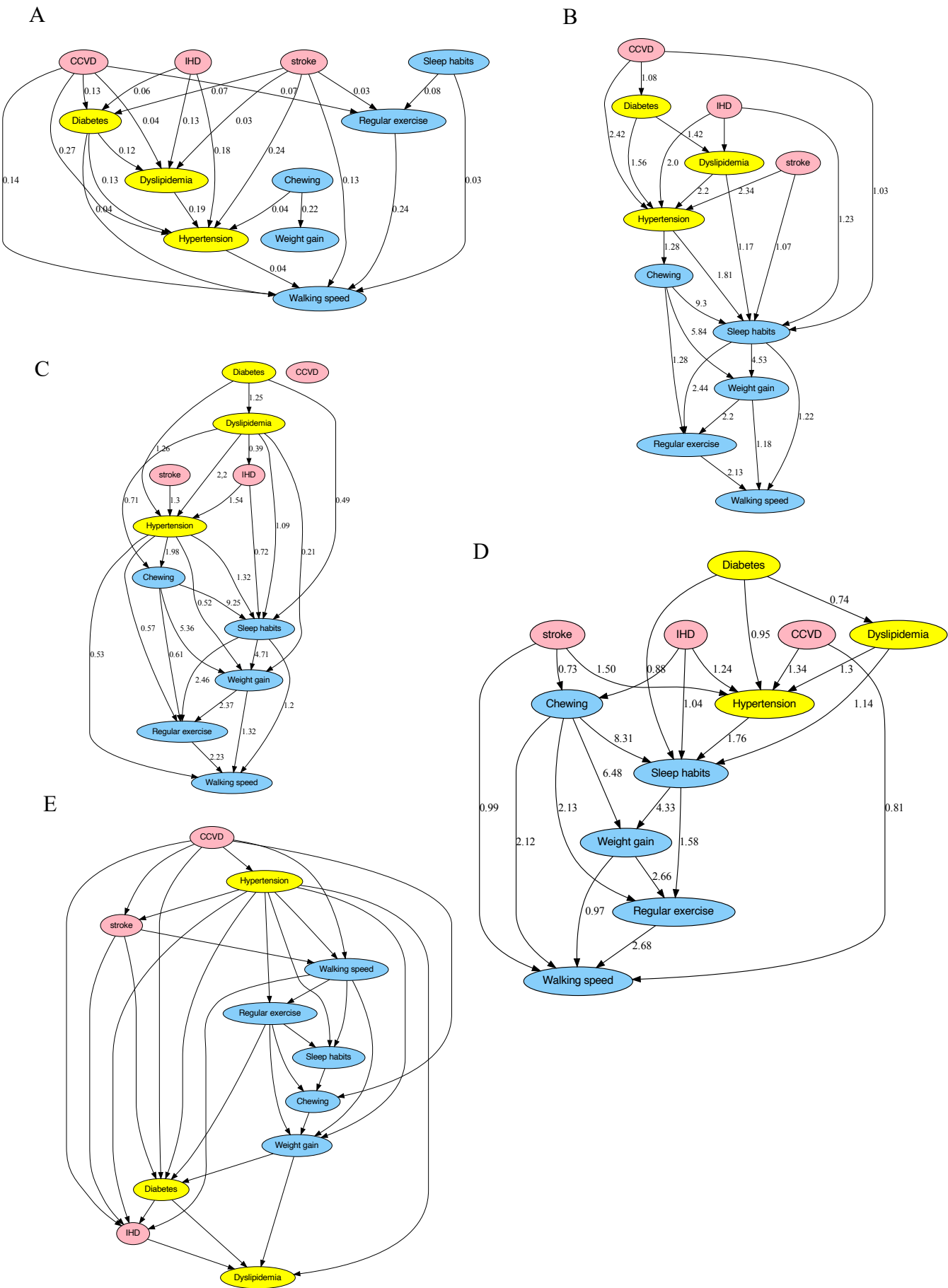

Supplement: Supplementary file 3 — Supplementary Figure S3. [file 41598_2024_61047_MOESM3_ESM.pdf]
