# Supplementary material for: Prediction and causal inference of cardiovascular and cerebrovascular diseases based on lifestyle questionnaires
Source: Sci Rep. 2024 May 7;14:10492. doi: 10.1038/s41598-024-61047-w (PMC11076536; doi:10.1038/s41598-024-61047-w)
Supplement: Supplementary file 4 — Supplementary Information. [file 41598_2024_61047_MOESM4_ESM.pdf]

別紙３

標準的な質問票

	質問項目	回答
1-3	現在、a からcの薬の使用の有無※①	
1	a.　血圧を下げる薬	①はい ②いいえ
2	b.　インスリン注射又は血糖を下げる薬	①はい ②いいえ
3	c.　コレステロール※②を下げる薬	①はい ②いいえ
4	医師から、脳卒中（脳出血、脳梗塞等）にかかっているといわれたり、治療を受けたことがありますか。	①はい ②いいえ
5	医師から、心臓病（狭心症、心筋梗塞等）にかかっているといわれたり、治療を受けたことがありますか。	①はい ②いいえ
6	医師から、慢性の腎不全にかかっているといわれたり、治療（人工透析）を受けたことがありますか。	①はい ②いいえ
7	医師から、貧血といわれたことがある。	①はい ②いいえ
8	現在、たばこを習慣的に吸っている。<br>（※「現在、習慣的に喫煙している者」とは、「合計 100 本以上、又は6ヶ月以上吸っている者」であり、最近 1 ヶ月間も吸っている者）	①はい ②いいえ
9	20 歳の時の体重から 10kg 以上増加している。	①はい ②いいえ
10	1 回 30 分以上の軽く汗をかく運動を週 2 日以上、1 年以上実施	①はい ②いいえ
11	日常生活において歩行又は同等の身体活動を 1 日 1 時間以上実施	①はい ②いいえ
12	ほぼ同じ年齢の同性と比較して歩く速度が速い。	①はい ②いいえ
13	この 1 年間で体重の増減が±3 kg以上あった。	①はい ②いいえ
14	人と比較して食べる速度が速い。	①速い ②ふつう ③遅い
15	就寝前の 2 時間以内に夕食をとることが週に 3 回以上ある。	①はい ②いいえ
16	夕食後に間食（3 食以外の夜食）をとることが週に 3 回以上ある。	①はい ②いいえ
17	朝食を抜くことが週に 3 回以上ある。	①はい ②いいえ
18	お酒（清酒、焼酎、ビール、洋酒など）を飲む頻度	①毎日 ②時々③ほとんど飲まない（飲めない）
19	飲酒日の１日当たりの飲酒量<br>清酒1合（180ml）の目安：ビール中瓶1本（約500ml）、焼酎35度（80ml）、ウイスキーダブル一杯（60ml）、ワイン2杯（240ml）	①1合未満 ②1～2合未満<br>③2～3合未満 ④3合以上
20	睡眠で休養が十分とれている。	①はい ②いいえ
21	運動や食生活等の生活習慣を改善してみようと思いますか。	①改善するつもりはない<br>②改善するつもりである（概ね6か月以内）<br>③近いうちに（概ね1か月以内）改善するつもりであり、少しずつ始めている<br>④既に改善に取り組んでいる（6か月未満）<br>⑤既に改善に取り組んでいる（6か月以上）
22	生活習慣の改善について保健指導を受ける機会があれば、利用しますか。	①はい ②いいえ

※①医師の診断・治療のもとで服薬中の者を指す。　※②中性脂肪も同様に取扱う。
