# Supplementary material for: Prediction and causal inference of cardiovascular and cerebrovascular diseases based on lifestyle questionnaires
Source: Sci Rep. 2024 May 7;14:10492. doi: 10.1038/s41598-024-61047-w (PMC11076536; doi:10.1038/s41598-024-61047-w)
Supplement: Supplementary file 5 — Supplementary Tables. [file 41598_2024_61047_MOESM5_ESM.docx]

**Supplementary Table 1.**

| rank | Feature | score |
| --- | --- | --- |
| 1 | Walking speed | 142 |
| 2 | Chewing | 100 |
| 3 | Weight gain | 85 |
| 4 | Sleep habits | 84 |
| 4 | Regular exercise | 84 |
| 6 | Alcohol consumption | 82 |
| 7 | Frequent snacking | 81 |
| 8 | Alcohol drinking frequency | 80 |
| 9 | Current smoking | 78 |
| 10 | Eating speed | 77 |
| 11 | Daily activity | 73 |
| 12 | Eating dinner late | 71 |
| 13 | Frequent skipping breakfast | 55 |

**Supplemental Table 2. Questionnaire list**

|  | Abbreviations | Lifestyle-related questions | Choice |
| --- | --- | --- | --- |
| 1 | Walking speed | Do you walk faster than your peers? | Yes or No |
| 2 | Chewing | How thoroughly do you chew your food during meals? |  |
| 3 | Weight gain | Have you gained 10 kg or more since turning 20 | Yes or No |
| 4 | Sleep habits | Do you feel you get sufficient sleep? | Yes or No |
| 5 | Regular exercise | Do you engage in at least 30 minutes of exercise twice a week for a minimum of one year? | Yes or No |
| 6 | Alcohol consumption | How often do you drink alcohol? | Every day,  Occasionaly, or  Almost never/Never |
| 7 | Frequent snacking | Do you consume food or beverages outside regular meals (breakfast, lunch, dinner)? | Yes or No |
| 8 | Alcohol drinking frequency | How much alcohol do you consume per session on drinking days? | Less than 1 cup of sake,  1-2 cup of sake,  2-3. cup of sake, or  More than 3 cup of sake, |
| 9 | Current smoking | Do you habitually smoke? | Yes or No |
| 10 | Eating speed | Do you eat more quickly than your peers? | Yes or No |
| 11 | Daily activity | Do you partake in at least one hour of physical activity daily at a walking pace? | Yes or No |
| 12 | Eating dinner late | Do you eat dinner within two hours of bedtime at least three times a week? | Yes or No |
| 13 | Frequent skipping breakfast | Do you skip breakfast at least three times a week? | Yes or No |
